# Finite Element Analysis of Bonding Property and Flexural Strength of WUHPC-NC Gradient Concrete

**DOI:** 10.3390/ma16103625

**Published:** 2023-05-09

**Authors:** Ziyang Tian, Rui Ma, Daosheng Sun, Wei Zhang, Aiguo Wang

**Affiliations:** 1 School of Materials and Chemical Engineering, Anhui Jianzhu University, Hefei 230022, China; tianzy1205@163.com (Z.T.);; 2 Anhui Province Engineering Laboratory of Advanced Building Materials, Anhui Jianzhu University, Hefei 230022, China

**Keywords:** gradient concrete, white ultra-high-performance concrete, bonding properties, flexural strength, finite element analysis

## Abstract

Ultra-high-performance concrete (UHPC) has greater mechanical and durability performance than normal concrete (NC). Using a limited dosage of UHPC on the external surface of NC to form a gradient structure could significantly improve the strength and corrosion resistance of the concrete structure and avoid the problems caused by bulk UHPC. In this work, white ultra-high-performance concrete (WUHPC) was selected as an external protection layer for normal concrete to construct the gradient structure. WUHPC of different strengths were prepared, and 27 gradient WUHPC-NC specimens with different WUHPC strengths and interval times of 0, 10, and 20 h were tested using splitting tensile strength to reveal the bonding properties. Fifteen prism gradient specimens with the size of 100 × 100 × 400 mm and a WUHPC ratio of 1:1, 1:3, and 1:4 were tested using the four-pointed bending method to study the bending performance of the gradient concrete with different WUHPC thicknesses. Finite element models with different WUHPC thicknesses were also built to simulate the cracking behaviors. The results showed that the bonding properties of WUHPC-NC were stronger with less interval time and reached the maximum of 1.5 MPa when the interval was 0 h. Moreover, the bond strength first increased and then decreased with the decline in the strength gap between WUHPC and NC. When the thickness ratios of WUHPC to NC were 1:4, 1:3, and 1:1, the flexural strength of the gradient concrete improved by 89.82%, 78.80%, and 83.31%, respectively. The major cracks rapidly propagated from the 2 cm position to the bottom of the mid-span, and the thickness of 1:4 was the most efficient design. The results simulated by finite element analysis also proved that the elastic strain at the crack propagating point was the minimum and was easier to crack. The simulated results were in good accordance with the experimental phenomenon.

## 1. Introduction

Concrete is the most popular construction material in use and is essential in civil engineering applications, such as infrastructures, buildings, bridges, and highways [1,2]. However, as an energy consumption material, the amount of concrete being used is growing every year; thus, how to extend the service life and reduce CO_2_ emissions during the full life cycle of concrete is a crucial problem that needs to be solved [3,4]. Concrete exposed to the environment is eroded by corrosive substances, for instance, chloride or sulfate ion corrosion, freeze–thaw damage, and carbonization. The corrosion process causes concrete deterioration and eventually failure earlier than its designed lifespan [5,6,7]. Ultra-high-performance concrete (UHPC) is a cementitious composite with excellent mechanical strength and better permeability resistance than normal concrete and is preferred for use in long-span bridges, buildings in severe environments, defense engineering, etc., and significantly increases the service life of building structures [8,9,10,11,12]. In addition, white UHPC (WUHPC) has not only inherited these excellent mechanical properties and durability but also enhances the aesthetic appearance of buildings with its unique visual impact. The lower absorption rate in terms of light and heat could reduce energy consumption and improve the green energy efficiency of buildings [13,14], and ecological WUHPC with a high fraction of admixture was studied in our previous work [15,16]. However, due to the requirement of a large fraction of fine reactive powders, the hydration of UHPC at an early age was intense, leading to high temperatures within the structure and significant autogenous shrinkage [17,18]. Meanwhile, the specific gravity and material costs of UHPC are far beyond normal concrete. These drawbacks led to UHPC being commonly used as a repair material to strengthen old NC structures rather than being widely used in practical applications [19,20,21,22]. In this situation, an investigation into how to use limited UHPC dosage to significantly improve the mechanical properties and durability of normal concrete is a feasible and potential way to promote the low-carbon development of concrete materials.

The functional gradient approach is a common method used to design materials with the desired performance and is often used in nanomaterial synthesis [23,24,25,26,27]. Although it is difficult to precisely control the properties of concrete, a gradient structure can also enhance the macro-performances with different components and mix proportions [28,29,30,31]. Several investigations have reported on UHPC-NC combined structures [32,33,34,35,36]. Zhang et al. [37] studied the NC-UHPC composite members in continuous box-girder bridges. Here, two 2200 mm long NC-UHPC composite columns consisting of UHPC and NC columns were experimentally tested, and the results showed that the creep coefficients of the NC-UHPC significantly decreased compared with NC beams. Lin et al. [34] explored the bending performance and cracks of railway T-beams composited with UHPC and NC. Three slab specimens with a cross-section of 300 × 600 mm and a height of 650 mm were prepared using UHPC and NC composites, and it was found that the strength and cracking moment was reduced in the composite structures. Some simulated models were also reported to reveal the bending performance and cracking of UHPC-NC composite structures [34,35,36]. Majid [38] developed an FE model to predicate the flexural behavior of UHPC-NC beams with four different steel fiber ratios, and the results revealed that, for a reinforcement ratio of 0.009, a hybrid UHPC-NC beam with only the middle 50% composed of UHPC achieved the same capacity as a beam made entirely of UHPC when the NC’s compressive strength was 30 MPa or more. By designing UHPC-NC composite beams of different NC thicknesses, Zhang [39] found that UHPC-NC and UHPC have the same carrying capacity when the thickness of NC is 60 mm, and the carrying capacity is the worst when the thickness of the NC is 100 mm, but the maximum bearing capacity is similar to that of the former at approximately 200 kN. The above research proved that when combined with UHPC, the mechanical properties of the NC structures were significantly improved. However, most research focused on beam or girder structures, and when such structures are composited with UHPC and NC, their scale becomes significantly larger. Research on urban architecture, especially on the improvement of the intrinsic performance of concrete materials, has rarely been reported.

The bonding property is one of the key points for the gradient concrete, which is affected by many factors, such as stiffness, roughness, moisture, and shrinkage [40]. Since the shrinkage of UHPC at an early age was much higher than NC, large internal stress was induced in the transition zone. Yuan et al. [41] summarized the bonding properties of UHPC-NC based on the reports. A total of 563 + 38 specimens were collected to reveal the factors that influenced interface strength in four aspects, and an artificial neural network was constructed to predict the bonding strengths. Yu et al. [42] roughed the interface of UHPC-NC to increase the bonding strength, and the results showed that the shear force increased by 202% and 121% when the roughness was 0.41 mm and 1.31 mm, respectively. Tian et al. [43] treated UHPC-NC with different methods and found that the shear strength at the joint surface increased by 82.17% once the surface was roughed by a groove. However, most works were aimed at fresh UHPC and old NC; the research on bonding strength between UHPC and NC before hardening was minimally reported. Still, in the gradient concrete, a unique transition zone formed when two concretes contacted and reacted together, and the bonding properties were mostly determined by the components and structures of this transition zone. However, the micro-properties of the transition zone in the gradient concrete have rarely been studied based on our survey.

**Figure 1:**
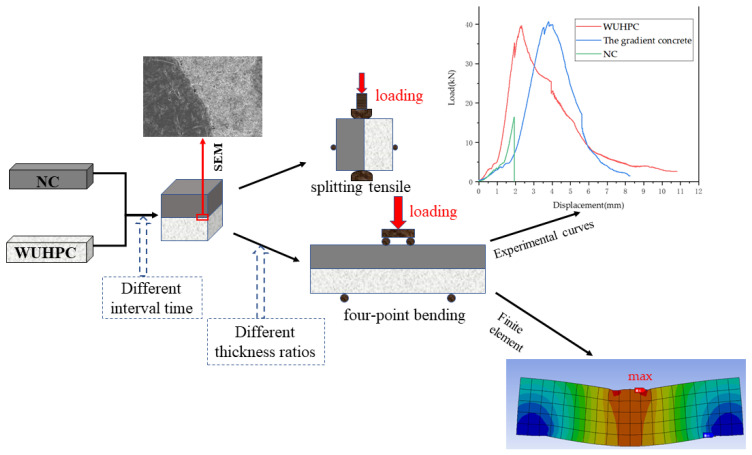
Schematic flow chart of the study.

In this work, a white UHPC (WUHPC)-NC gradient concrete was constructed to significantly improve the mechanical strength, corrosion resistance, and aesthetic appearance with a small dosage of WUHPC so that this gradient concrete could be widely used in urban buildings, city overpass bridges, and landscape architectures. WUHPC was designed as a thin overlayer to control the dosage and protect the inner NC from an exposed environment. The interval time of two concretes, and the strength degree of WUHPC were varied to study the bonding performance of the gradient concrete to determine the optimal mixed ratio and formation technology. The bending properties of the WUHPC-NC gradient concrete were also evaluated by flexural strength and four-pointed bending. Finite element analysis was used to reveal the strain distribution in the concrete and to predict the crack position. The research steps are illustrated in Figure 1.

## 2. Materials and Methods

### 2.1. Materials

**Figure 2:**
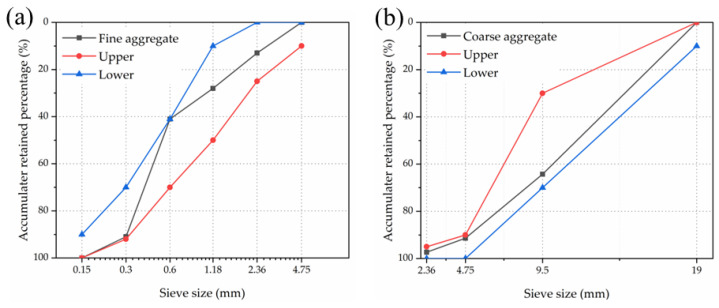
The grading curve of (**a**) fine aggregate and (**b**) coarse aggregate.

**Figure 3:**
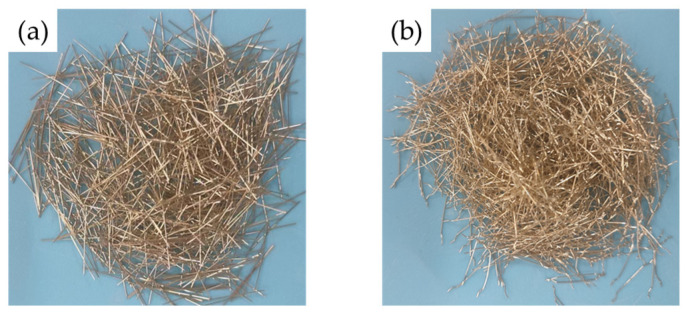
Image of steel fibers: (**a**) straight and (**b**) hooked.

P·W 52.5 White Portland Cement (WPC), P·O 42.5 Portland Cement (PC), white silica fume (WSF), metakaolin (MK), limestone powder (LP), and Fly ash (FA) were selected as binder materials. The physical properties and the chemical compositions of the binders are shown in Table 1 and Table 2, respectively. Fine aggregate was river sand with a fineness modulus of 2.59. Coarse aggregate was stone with continuous grade from 5 to 19 mm. The gradation curves of the fine and the coarse aggregate are shown in Figure 2; both aggregates were in good gradation. The polycarboxylate water-reducing agent (WR) was solid powder with a reducing range of about 30% and was added to improve the workability. Straight (SSF) and end-hooked (HSF) steel fibers with a length of 13 mm and an aspect ratio of 65 were used as the reinforced material; the morphology of steel fibers is shown in Figure 3.

### 2.2. Mix Proportions and Process

To reveal the effect of the strength gap on the bonding performance of the gradient WUHPC-NC, the WUHPC with different strengths was prepared in this experiment. The WUHPC was prepared by WPC, LP, WSF, MK, and sand without coarse aggregate. The strength of WUHPC was adjusted by W/B ratio, mineral admixture substituted ratio, and fiber content, while the hybrid fiber ratio was kept with SSF:HSF = 2:1. The detail of mix proportions and the designed strength of WUHPC and NC are listed in Table 3.

For WUHPC preparation, powder materials, including WPC, WSF, MK, LP, and sand, were first dry mixed in a container, then water and WR were added with continuous stirring until the uniform matrix was formed. Last, steel fibers were slowly added in 2–3 min. The preparation method of NC was traditional; the powders and stones were mixed with water in a blender.

**Figure 4:**
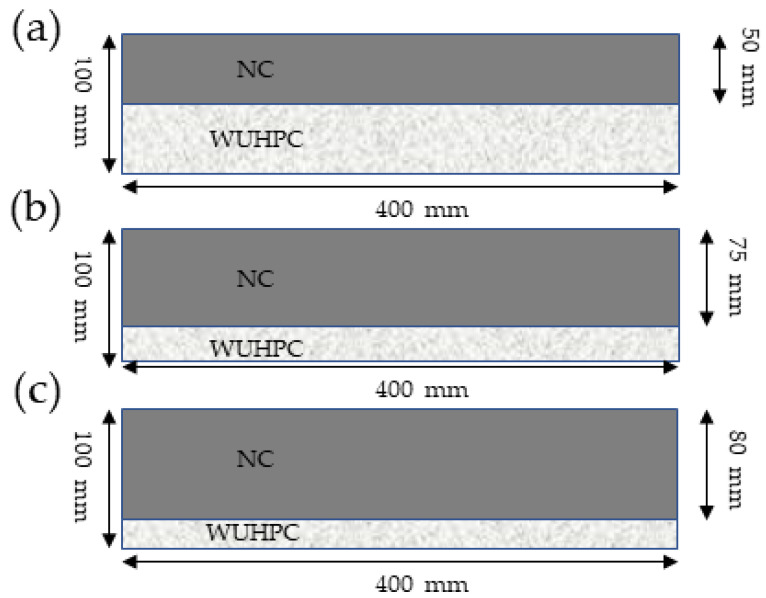
Image of the different thickness ratios of WUHPC to NC: (**a**) 1:1; (**b**) 1:3; (**c**) 1:4.

To form the gradient concrete, the prepared WUHPC and NC were cast successively into the mold of 100 × 100 × 100 mm for the splitting tensile strength test and 100 × 100 × 400 mm for the bending strength test. Twenty-seven samples were used in the splitting tensile strength test, and 15 samples were used in the bending test in total. Three different interval times between WUHPC and NC casting were chosen, which were 0 h, 10 h, and 20 h as a comparison. Additionally, the thickness ratios of WUHPC to NC of 1:1, 1:3, and 1:4 were prepared to reveal the optimal dosage of WUHPC, which is shown in Figure 4. After cast, the gradient specimens were placed under standard conditions (25 °C, RH > 95) for 1.5 d, then de-molded and steam curried at 85 °C for 3 d.

### 2.3. Experimental Method

#### 2.3.1. Splitting Tensile Strength

**Figure 5:**
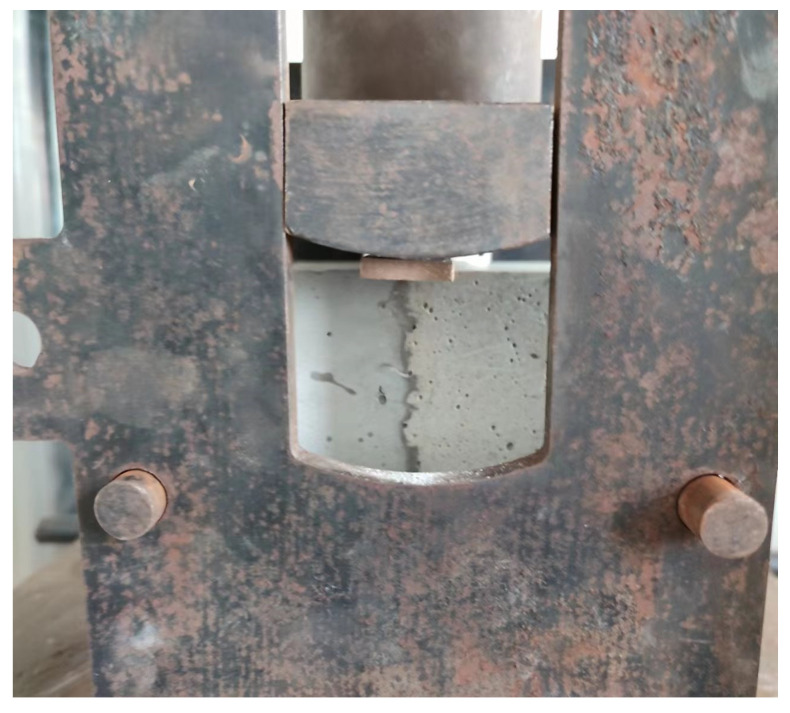
Setup of the splitting strength testing.

The bonding property between WUHPC and NC in the gradient concrete was characterized by the splitting tensile strength. During the test, the specimen was pleased in the mold, with the interface verticalness. Two steel sticks were put at the junction of two materials; then, a vertical load was applied to the steel sticks at the rate of 0.05 MPa/s. The experimental setup is shown in Figure 5. When the load was greater than the bonding of the gradient concrete, WUHPC and NC were split into two parts from the joint surface. When the specimen was split, the load was recorded and calculated to the splitting tensile strength.

#### 2.3.2. Bending Strength

**Figure 6:**
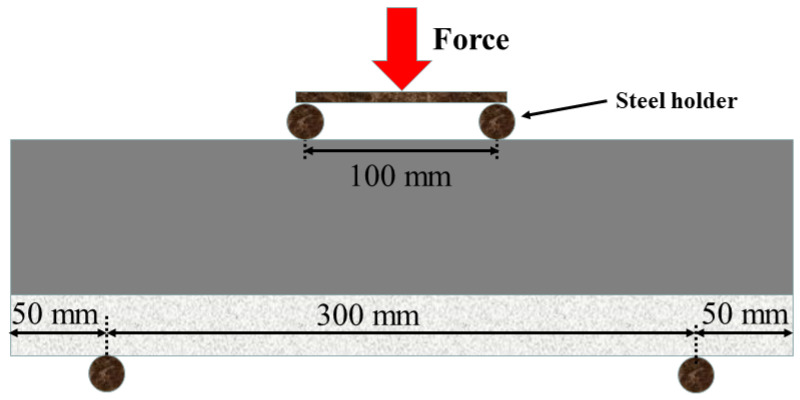
Setup of the flexural strength testing.

According to standard ASTM C1069 [40], a universal testing machine was used to test the bending strength by the four-point bending method. The specimen size was 100 × 100 × 400 mm, with the thickness ratio of WUHPC: NC = 1:1, 1:3, and 1:4, respectively. In the test, the NC part was settled upside, with a span length of 100 mm, and the WUHPC part was at the bottom, with a span length of 300 mm. The sketched setup is shown in Figure 6. When the test began, a vertical load with a speed of 0.05 mm/s was applied from the top, and the deflection of the specimen with time was recorded. A stress-strain curve was obtained after the failure of the specimen.

#### 2.3.3. Compressive Strength

**Figure 7:**
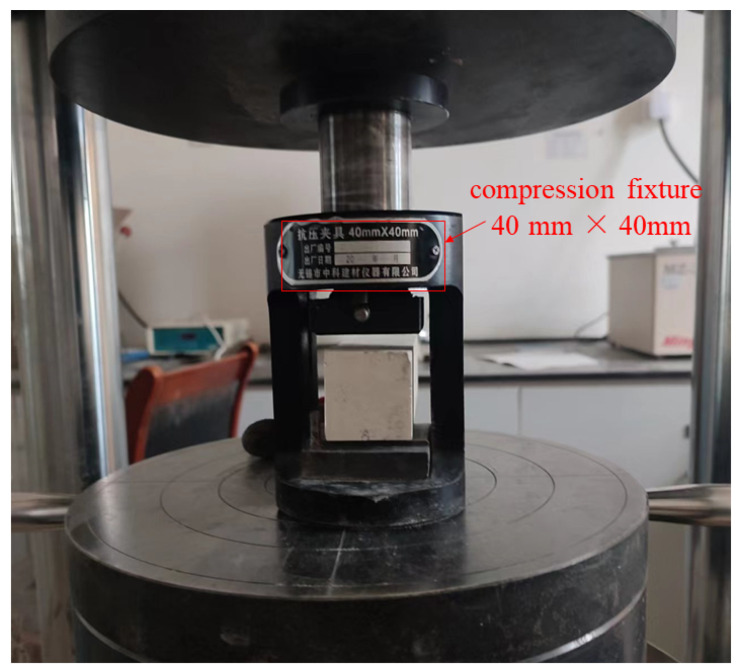
Setup of the compressive strength test for WUHPC.

The specimens of NC and WUHPC with the size of 100 × 100 × 100 mm and 40 × 40 × 160 mm were used for compressive strength testing, and the load rates applied for two concretes were 0.5 MPa/s and 2.5 MPa/s, respectively. For each group, three specimens were tested, and the average results were calculated as the compressive strength. Figure 7 is the setup of the compressive strength test for WUHPC. The compressive load was stopped when WUHPC failed, and the maximum load was recorded and calculated as the compressive strength.

#### 2.3.4. Microstructure

The morphology and the element distribution of the gradient concrete at the joint region were analyzed by scanning electron microscopy (SEM). The specimens around the joint area were cut into slices with a thickness of 5 mm by a high-speed precision cutting machine. Then, the test slices were immersed in anhydrous ethanol to stop hydration.

## 3. Results and Discussion

### 3.1. Splitting Tensile Strength

**Figure 8:**
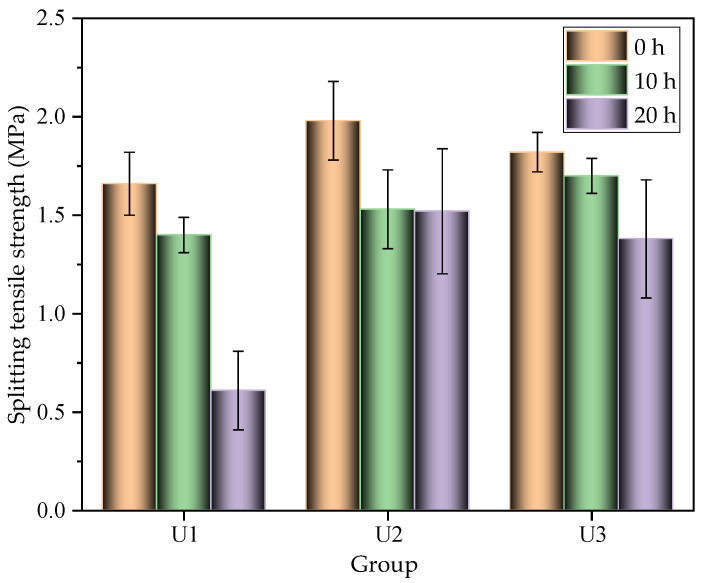
Splitting tensile strengths of the gradient concrete for different interval times.

Figure 8 shows the splitting tensile strength of the gradient concrete with different combined methods. It was found that with the increase of interval time, the splitting tensile strength decreased for all groups. The strength reached the maximum, approximately 2.0 MPa, once the interval time was 0 h. Still, when the strength difference between WUHPC and NC was the highest, the splitting tensile strength was the minimum. It first increased and then decreased with the decrease of WUHPC strength. Taking the interval time of 0 h as an example, the splitting tensile strengths of U1, U2, and U3 were 1.66 MPa, 1.98 MPa, and 1.82 MPa, respectively, which were close to the results by Fan [44]. Therefore, when the interval time was 0 h, the bond performance between WUHPC and NC was the best. The short interval time was equivalent to the rough treatment at the interface.

The cementitious materials in concrete reacted to harden with time; it was easily understood that shorter interval time resulted in a longer reaction time of two concretes and led to stronger bonding strength. Once WUHPC and NC were cast together with an interval time of 0 h, the contact region was fresh and blended to react continuously and form a complete block. When the interval time increased, the activity of cementitious materials declined, and the surface began to lose plastic characteristics after the initial setting time so that an obvious boundary was generated at the interface between two concretes, which would weaken the bonding property. Additionally, with the strength increased, the matrix of WUHPC became denser, and the fresh NC was hard to mix at the interfacial position, resulting in a lower splitting tensile strength. Furthermore, compared with U2 and U3, more steel fiber was used in U2, the bonding property of the interface was better than in U3, and the splitting tensile strength was also increased.

**Figure 9:**
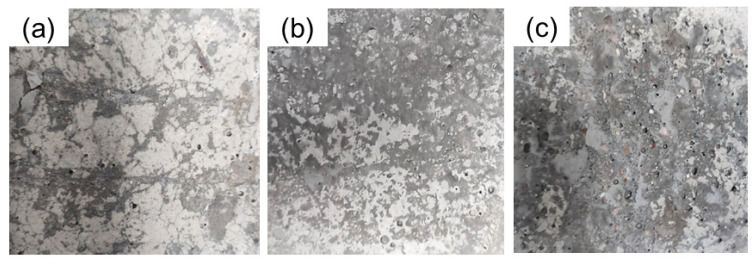
Split surface morphologies of the gradient concrete: (**a**) U1; (**b**) U2; (**c**) U3.

**Figure 10:**
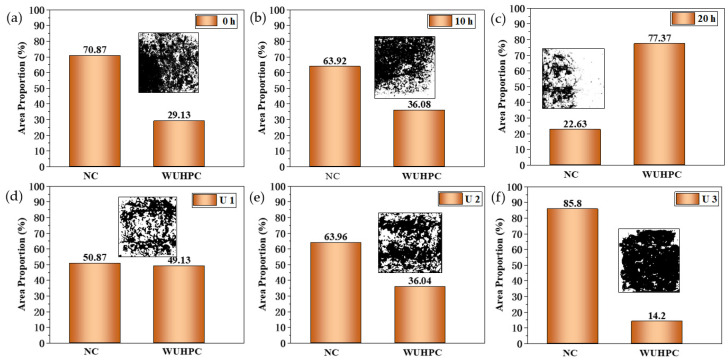
Area proportion of NC adhered on splitting surface of (**a**) 0 h; (**b**) 10 h; (**c**) 20 h; (**d**) U1; (**e**) U2; (**f**) U3.

The morphologies of the split surface of the gradient concrete samples after the test are shown in Figure 9. The white part was the WUHPC matrix, while the gray part was the adhered mortar of NC. More mortar of NC adhered at the interface, better bonding performance of the gradient concrete. To quantitatively compare the ratio of adhered NC with different combined processes, the digital pictures were binarized, and the area ratio of NC coved on WUHPC is statistically summarized in Figure 10.

In Figure 10, the black binarization picture referred to NC, while the blank was WUHPC. The proportion of NC also reflected the bonding performance of the WUHPC–NC interface. It was found that the ratio of NC decreased with the longer interval time and sharply decreased when the interval time increased to 20 h. This result corresponded to the splitting tensile strength. A shorter interval time led to a full mix reaction between NC and WUHPC, resulting in a better bonding property. However, the adhered NC was varied with different WUHPC strengths. With the WUHPC strength of 192. 1, 157.6, and 126.0 MPa, the proportion of NC was 50.87%, 63.96%, and 85.80%, respectively. This might be because the higher strength of WUHPC would make the matrix denser, and NC was hard to blend into WUHPC. When the strength decreased to 126.0 MPa, almost the whole surface was covered with NC. In other words, the splitting happened in the NC matrix. In this case, the bonding strength is much more dependent on the materials of NC, not the transition interface. While U2 and U3 had the same W/B, the former contained more steel fibers. The bridging effect of steel fibers might be the reason why the bond performance of U2 was greater than U3.

### 3.2. Bending Performance

The flexural strength of U2, NC, and the gradient concretes with different WUHPC thicknesses are summarized in Table 4. The flexural strength of NC was 5.99 MPa, while for WUHPC was 11.87 MPa, which was twice as high as the former. When the thickness ratios (WUHPC:NC) were 1:4, 1:3 and1:1, the flexural strength compared with NC was increased by about 89.78%, 103.59%, and 95.83%, respectively, which were slightly vibrated nearby WUHPC.

The flexural strength was mostly determined by the ultimate load capacity under four-point bending. The results showed that when the thickness of WUHPC: NC = 1:3, the gradient concrete could bear the highest bending load. This proved that even with a small dosage of WUHPC, the gradient structure also performed an equivalent mechanical behavior to WUHPC under bending conditions.

**Figure 11:**
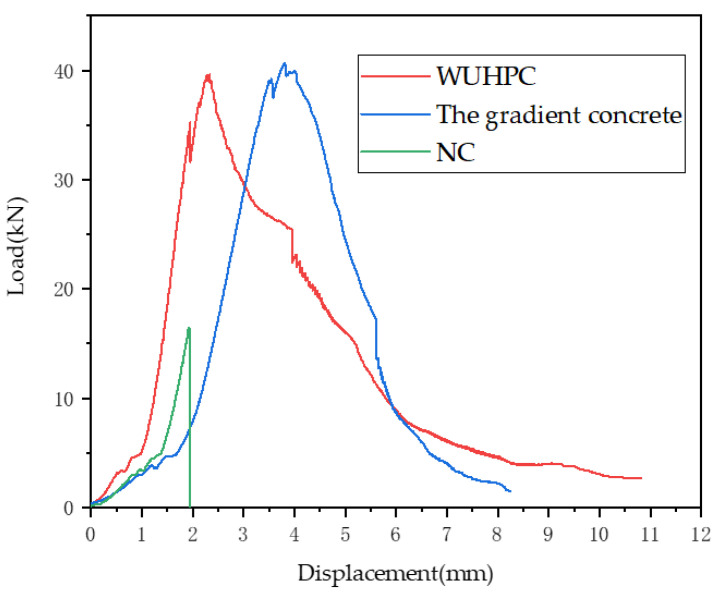
Force-displacement curves of the gradient concrete, WUHPC, and NC.

Figure 11 shows the force-displacement curves of the gradient concrete (1:3), WUHPC, and NC. From the curves, the ultimate load of WUHPC was much higher than that of NC. The NC specimen was directly broken at the ultimate load, while the deflections of WUHPC and the gradient concrete were still increased in a high range after the ultimate load. Still, for the gradient concrete, the displacement was even larger than WUHPC before the ultimate load point, then the increased rate was lower than WUHPC. The calculated toughness of WUHPC and the gradient concrete were 6.2 and 6.1, respectively, which were much more approximate. The maximum load of UHPC–NC was close to that of UHPC, which indicated that the addition of UHPC could effectively improve the carrying capacity of UHPC–NC, and the maximum load did not increase significantly with the increased thickness of UHPC. 

Due to the high strength and the reinforcement of fiber, the deformation of WUHPC was much better than NC, and it did not rupture after the ultimate load. With the WUHPC dosage of 1/3, the gradient concrete showed similar deformation properties with pure WUHPC.

**Figure 12:**
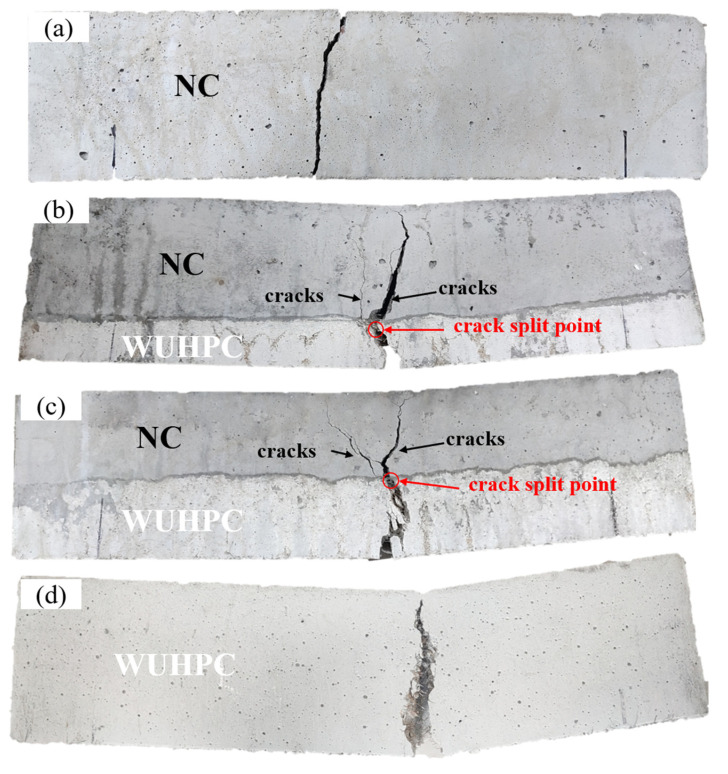
Photos of cracks in the gradient concrete: (**a**) NC; (**b**) WUHPC:NC = 1:3; (**c**) WUHPC:NC = 1:1; (**d**) WUHPC.

The photos of cracks in the gradient concrete after four-point bending tests are present in Figure 12. During the test, a main crack was generated near the mid-span at the bottom for all specimens and developed upward with the load increasing. For NC, this main crack rapidly developed throughout the specimen with little deformation. However, in WUHPC, the crack width was much larger than that in NC due to the bridge effect of steel fiber. In addition, when the cracks developed to the interface of the gradient concrete, more cracks were formed in the NC part, as seen in Figure 12b,c.

In sum, the mechanical strength of the gradient concrete composited with a small dosage of WUHPC was more closed to that of the pure WUHPC and was much better than NC. When the gradient concrete with a thickness ratio of 1:4 between WUHPC and NC was used in the construction, the mechanical strength was significantly improved with limited materials cost increase, and this might be accepted in field applications.

### 3.3. Microstructure of Bonding Surface

**Figure 13:**
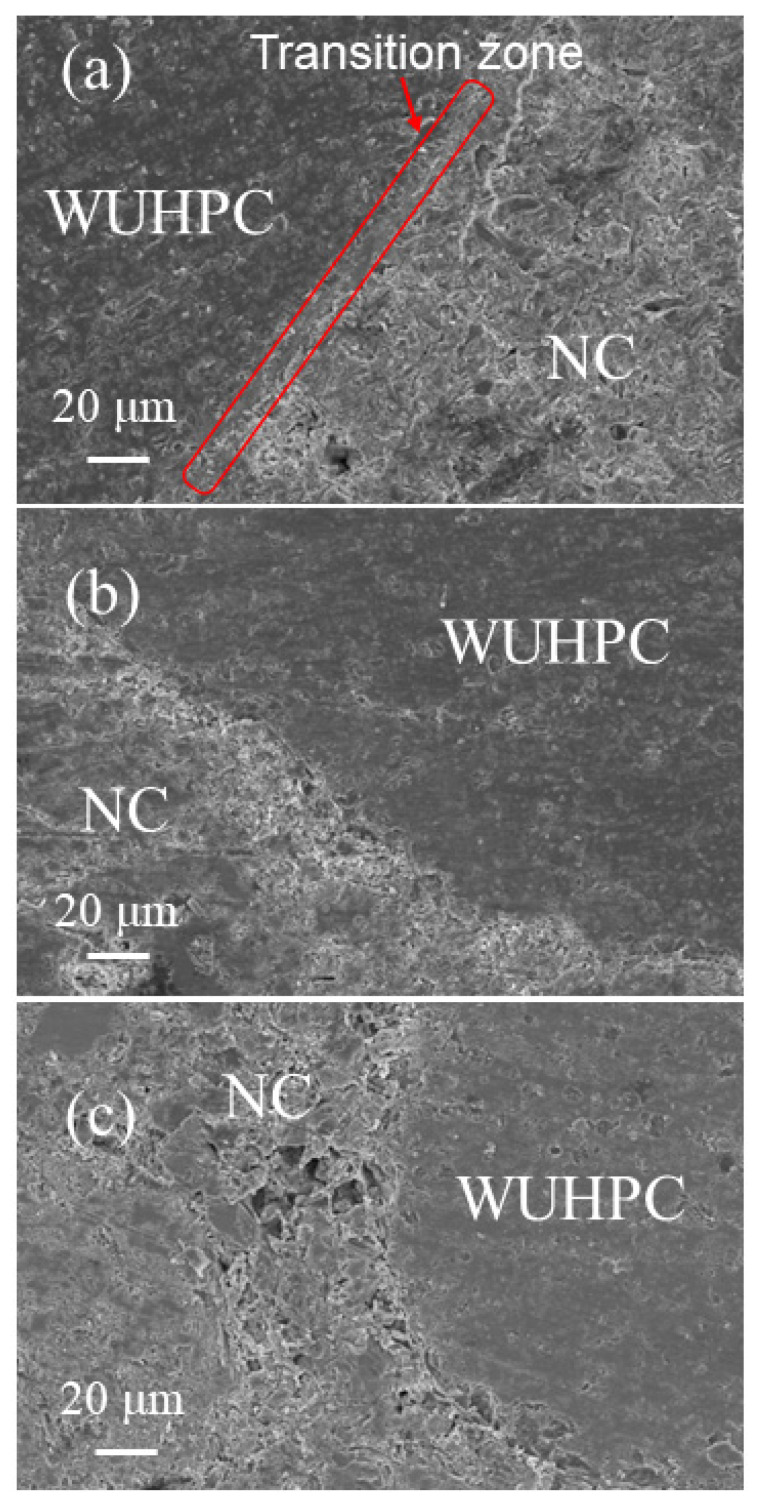
Microstructure at the junction of gradient concrete with different interval times: (**a**) 0 h; (**b**) 10 h; (**c**) 20 h.

The morphology of the gradient concrete around the contacted area is shown in Figure 13. From the pictures, the two concretes were easily distinguished. The matrix of WUHPC was much denser, with rare cracks and voids, while the NC was loose. However, once the interval time was 0 h, there was a transition zone formed between two concretes, as marked with a red frame in Figure 13a. The matrix in the transition zone was almost continuously changed from WUHPC to NC without defects. When the interval time increased to 10 h, and even longer, the transition zone was hard to find in images; instead, with the boundary between two concretes, the cracks and defects were highly increased.

**Figure 14:**
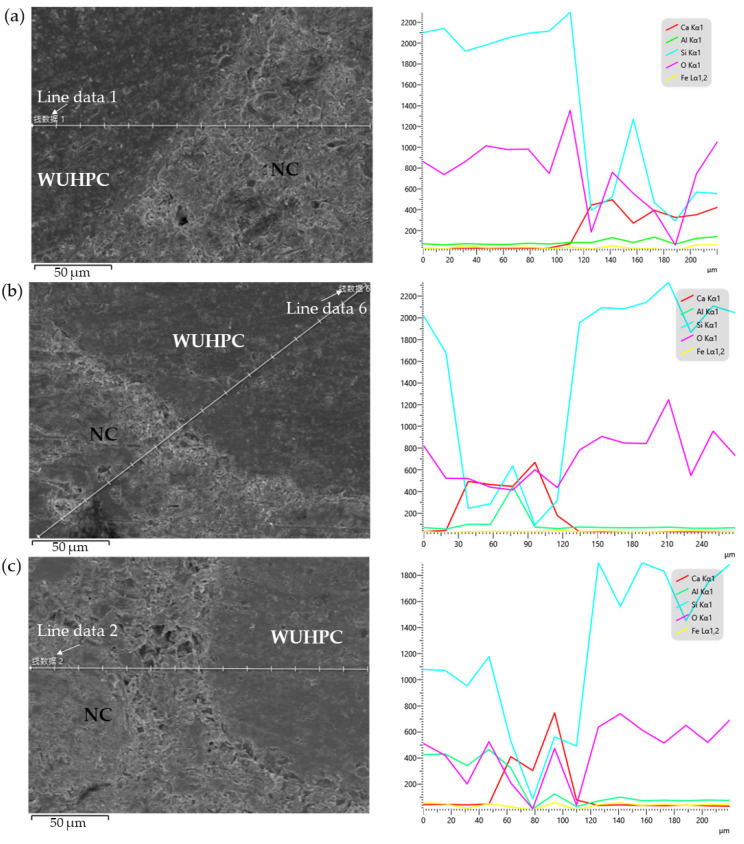
The energy spectrum line scanning and element distribution of the gradient concrete: (**a**) 0 h; (**b**) 10 h; (**c**) 20 h.

To analyze the difference between the two concretes, the element mapping crossing the boundary was tested and shown in Figure 14. In WUHPC, more Si and Al were detected due to the high fraction of mineral admixtures, such as WSF and MK. In contrast, the lower dosage of cement resulted in a lower content of Ca than NC. In Figure 14, the content of Si, Al, and Ca was reversed with the distance increased, and the reversion point reflected the boundary between WUHPC and NC. However, in Figure 14a, it was found that ranging in 120~180 μm, the content of Si was higher than that in NC, showing a slow decline. This phenomenon was not found in other samples. This also proved that a transition zone was formed with an interval time of 0 h, and once the time increased to 10 h, NC and WUHPC were hard to blend together.

The morphology and the linear element mapping of the matrix proved that there was indeed a transition zone in the gradient concrete, but the width of this zone was decreased with the increase of interval time. With the interval time of 0 h, two pieces of concrete were fresh and well-flowed. When they come into contact together, the matrix at the interface may blend into the other part due to gravity. That is why the elements Si and Ca vibrate at distances 120–160 μm in Figure 14a. As time went on, the blending effect became weaker, and the boundary of the two concretes was more obvious.

## 4. Finite Element Analysis

### 4.1. Finite Element Modeling

**Figure 15:**
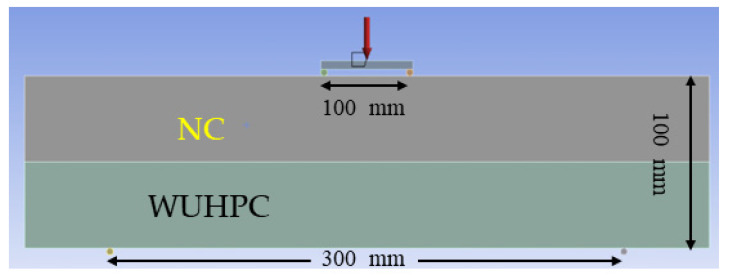
Finite element modeling.

The size of the finite element modeling was 100 × 100 × 400 mm, which was composed of different thicknesses of WUHPC. The thickness ratios of WUHPC and NC were 1:4, 1:3, and 1:1, respectively. The contact mode between WUHPC and NC was binding, which was perfect bonding. The support sticks were rigid without displacement change. The mesh size was set to 20 mm, shown in Figure 15. The load ratio and direction were the same as in the experiment. The elastic modulus and Poisson’s ratio of two concretes were determined from experiment and input as the simulation parameter; the results are listed in Table 5.

### 4.2. Total Deformation

**Figure 16:**
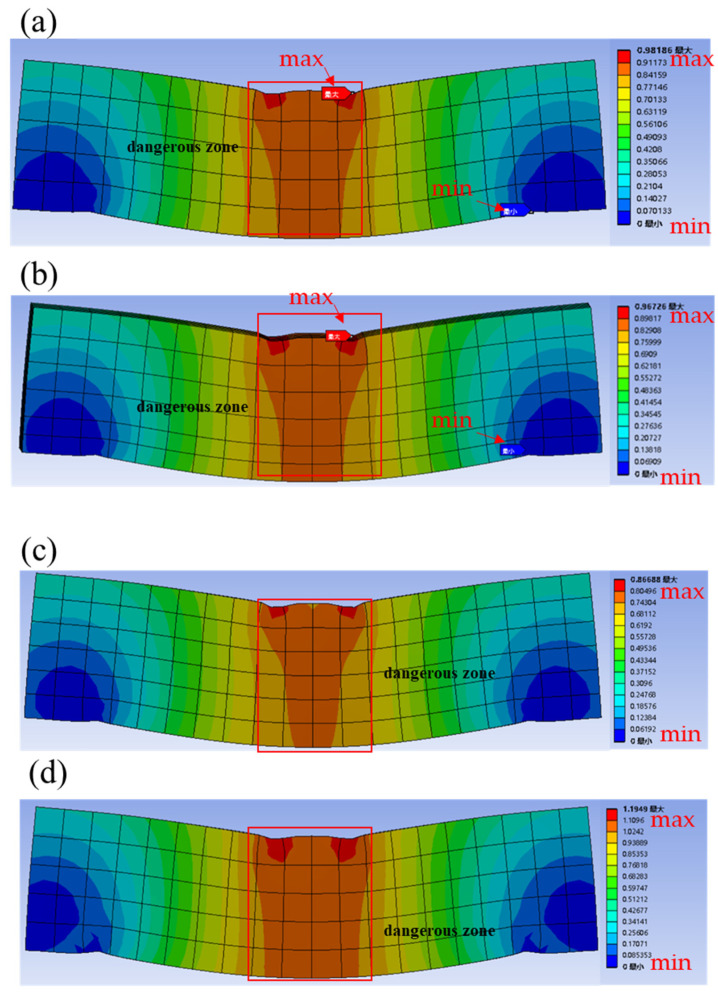
Total deformations of the gradient concrete: (**a**) WUHPC:NC = 1:4; (**b**) WUHPC:NC = 1:3; (**c**) WUHPC:NC = 1:1; (**d**) NC.

The total deformation refers to the amount of displacement generated when a structure is subjected to load, which reflects the stiffness of the structure. Figure 16 shows the total deformation of the gradient concrete. The dangerous zone of the gradient concrete and NC were mainly in the middle position, but with the increase of the thickness of WUHPC, the dangerous zone at the bottom of the middle span gradually shrunk, which indicates that WUHPC effectively inhibited the deformation of the gradient concrete. The maximum deformation was between the middle bottom and the loading area, so the cracks started from the bottom, which was consistent with the experiment phenomenon under four-point bending. After comparison, it was found that the total deformation of NC was the largest at 1.949 mm. With the increased thickness of WUHPC in the gradient concrete, the total deformation decreased gradually. Especially when WUHPC:NC = 1:4 or 1:3, as presented in Figure 16a,b, the difference in total deformation was 0.0146 mm, which was negligible. However, when the thickness of WUHPC continued to increase, the reduction rate of the total deformation declined, indicating that the improvement of the bending property of the gradient concrete dropped with the increase of WUHPC thickness. The addition of WUHPC could effectively reduce the range of dangerous areas in the middle span of the gradient concrete to reduce the damaged area in the bending resistance of the gradient concrete. Therefore, the cracks development of the gradient concrete mainly occurred in the middle span, which corresponds to the crack location in Figure 12.

Table 6 shows the total deformations of the gradient concrete at the intermediate position under four-point bending. It was clear that the deformation difference of NC between the bottom and center was the largest at 0.0205 mm. When WUHPC was added, the thickness ratios of WUHPC to NC were 1:4, 1:3, and 1:1; the deformation differences were 0.01437 mm, 0.1144 mm, and 0.00895 mm, respectively. The deformation difference between the bottom and center of the gradient concrete dropped with the thickness increased. This indicated that WUHPC could reduce the deformation of the gradient concrete, which proved the feasibility of the gradient concrete.

### 4.3. Elastic Strain

**Figure 17:**
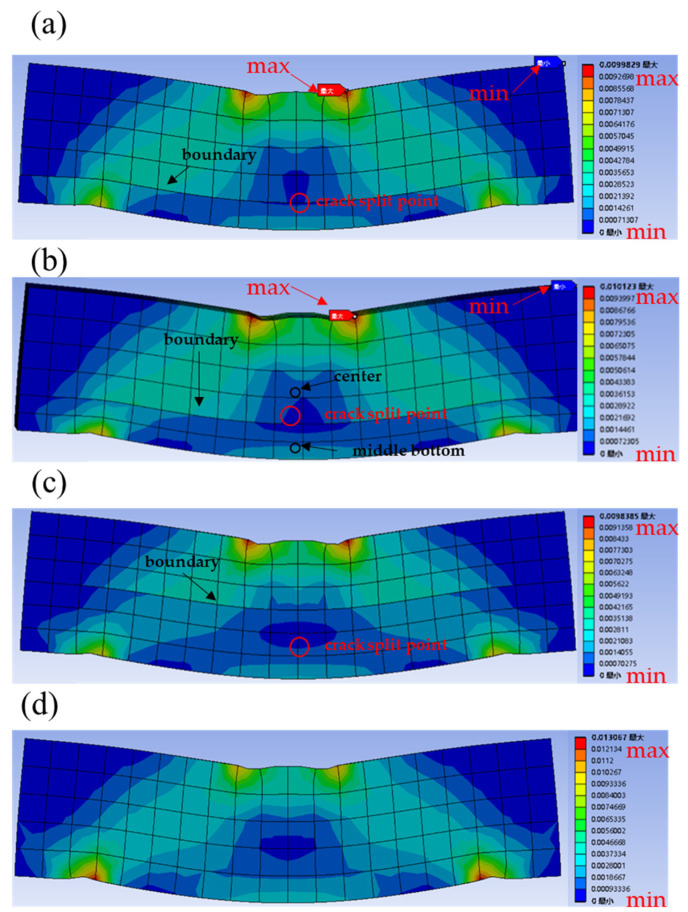
Equivalent elastic strains of the gradient concrete: (**a**) WUHPC:NC = 1:4; (**b**) WUHPC:NC = 1:3; (**c**) WUHPC:NC = 1:1; (**d**) NC.

The equivalent plastic strain was used to determine the position of the yield surface once the material was strengthened. Figure 17 is the equivalent elastic strain of the gradient concrete. It was observed that the equivalent elastic strain of the gradient concrete with different WUHPC thicknesses was analyzed by applying the same pressure to the loading plate. The equivalent stress in the WUHPC was mainly concentrated at the middle bottom and the support point, which indicated that the gradient concrete was relatively easy to fail from the bottom. Additionally, the equivalent elastic strain at the splitting point was obviously greater than those on both sides, which indicated that both sides were more vulnerable to failure, so the cracks split at this time. It was consistent with the experimental results. What is more, the equivalent elastic strains on both sides of the gradient concrete gradually decreased with the increase of the WUHPC thickness, and the safety zone gradually developed towards the middle, which indicated that WUHPC played a certain role in protecting NC during the bending test.

The total deformations and equivalent elastic strains of the gradient concrete were simulated and analyzed by finite element analysis. It was found that when the gradient concrete was subjected to four-point bending, the displacement and the strain at the center part were the largest, so the cracks usually started from the bottom of the middle part. When the pressure was constant, the total deformation of the gradient concrete decreased with the increase of WUHPC thickness, but the total deformation reduction rate also decreased. By comparing the equivalent strain contours of the gradient concrete, it could be found that there was a region on the upper side of the split point of the gradient concrete where the strain was the minimum, which was why the split occurred.

## 5. Conclusions

The combination of WUHPC and NC in the gradient concrete possessed better bending strength than plant NC. By studying the influence of pouring technology on the bonding property of the gradient concrete and the influence of WUHPC thickness on the flexural property of gradient concrete, the following conclusions are drawn through the numerical simulations by finite element analysis.

(1) The splitting tensile strength of the gradient concrete increased with the shorter interval time and reached the maximum once the strength gap between WUHPC and NC was moderate. With the increase of interval time, the boundary of the gradient concrete at the joint surface became more and more clear, which was not conducive to the bonding surface.
(2) WUHPC could effectively improve the flexural performance of the gradient concrete, but this effect was not linearly increased with the thickness of WUHPC. When the thickness ratios of WUHPC to NC were 1:4, 1:3, and 1:1, compared with NC, the flexural strength of the gradient concrete increased by 89.82%, 78.80%, and 83.31%, respectively. From the results, the small dosage of WUHPC could significantly improve the bending properties of NC.
(3) In the bending process, WUHPC was stressed first, and the cracks developed upward from the middle bottom of WUHPC, but the total development was relatively slow. Therefore, the deformation of the gradient concrete increased in the early stage, but when NC was stressed, the crack development speed increased. Moreover, there was a crack-splitting point that was independent of the thickness of WUHPC. In the experiment, the optimum thickness ratio (WUHPC:NC) of the gradient concrete was 1:4.
(4) The total deformation and equivalent elastic strain of WUHPC with different thicknesses under the same pressure were simulated by finite element analysis. The results showed that the damaged area of the gradient concrete was easy to occur in the middle position, which was consistent with the cracking position of the gradient concrete. The equivalent elastic strain at the crack splitting point was smaller than that around it, which resulted in easy cracking, and the simulated results were close to the experiment.
(5) By comparing the deformations of the middle bottom, the joint surface, and the center position, the displacement difference between the bottom and the middle section gradually decreased with the increase of WUHPC thickness. Therefore, the thickness of WUHPC was not suggested to be too large.
(6) The steam curing method was adopted for the gradient concrete in this study. Although the curing time was greatly shortened, the steam curing condition could not make the gradient concrete be formed on a large scale, which was not conducive to its application in practical engineering. At the same time, due to the difference between WUHPC and NC, steam curing would promote hydration, leading to a large shrinkage stress, which might not be conducive to the bond performance of the gradient concrete.

## Figures and Tables

**Table 1:** Physical properties of materials.

Materials	Specific Surface Area (m^2^/kg)	Density (g/m^3^)	Water Requirement (%)
WPC	420	3.02	29
WSF	22,800	2.20	-
MK	13,700	2.58	-
LP	970	2.80	-
PC	350	3.03	-
FA	270	2.78	-

**Table 2:** Chemical compositions of the cement and mineral admixtures (wt/%).

Materials	CaO	SiO_3_	Al_2_O_3_	Fe_2_O_3_	MgO	Na_2_O	SO_3_
WPC	67.09	18.09	2.25	0.27	4.49	0.30	4.33
WSF	0.29	91.83	0.48	0.40	0.33	0.10	0.97
MK	0.48	95.62	0.90	0.06	0.37	0.04	2.34
LP	99.36	0.22	0.02	0.03	0.28	-	0.01
PC	57.87	22.19	8.59	3.12	2.59	0.27	3.28
FA	4.01	50.66	30.83	6.07	0.92	0.89	1.68

**Table 3:** Mix proportions in kg/m^3^ and mechanical properties of WUHPC and NC.

Group	W/B	PC	WPC	FA	LP	WSF	MK	Water	Total Binder	WR	Sands	Stones	Fibers	Fiber Volume Content (vol%)	Strength (MPa)
U1	0.16	-	651.0	-	390.6	195.3	65.1	208.3	1302.0	18.2	1302.1	-	246.1	3%	192.1
U2	0.17	-	651.0	-	520.8	65.1	325.5	265.6	1562.4	18.2	1302.1	-	246.1	3%	157.6
U3	0.17	-	651.0	-	520.8	65.1	325.5	265.6	1562.4	18.2	1302.1	-	122.4	1.5%	125
C40	0.40	576.0	-	135.5	-	-	-	284.6	711.5	-	1000.0	1150.0	-	-	48

Note: The strength in the table is obtained after three days of steaming at 85 °C.

**Table 4:** Flexural strength of the gradient concretes with different thickness ratios.

Thickness Ratios	Flexural Strength (MPa)	Increased Ratio
NC	5.99	0
WUHPC	11.87	98.16%
WUHPC:NC = 1:4	11.37	89.79%
WUHPC:NC = 1:3	12.20	103.59%
WUHPC:NC = 1:1	11.73	95.83%

**Table 5:** Finite element simulation parameter.

Material	Modulus of Elasticity (Pa)	Poisson’s Ratio
NC	2.4 × 10^10^	0.17
WUHPC	4 × 10^10^	0.2
Rigid sticks	2.0 × 10^11^	0.3

**Table 6:** Total deformations of the gradient concrete with different WUHPC/NC ratios.

	Bottom	Junction Surface	Center
NC	1.0771 mm	-	1.0975 mm
WUHPC:NC = 1:4	0.86164 mm	0.86817 mm	0.87601 mm
WUHPC:NC = 1:3	0.848 mm	0.85508 mm	0.85944 mm
WUHPC:NC = 1:1	0.75058 mm	0.75953 mm	0.75953 mm

## Data Availability

Not applicable.
